# DOES SIZE MATTER? A CRITICAL REVIEW OF META-ANALYSIS IN AGRONOMY

**DOI:** 10.1017/S0014479719000012

**Published:** 2019-03-14

**Authors:** TIMOTHY J. KRUPNIK, JENS A. ANDERSSON, LEONARD RUSINAMHODZI, MARC CORBEELS, CAROL SHENNAN, BRUNO GÉRARD

**Affiliations:** † International Maize and Wheat Improvement Centre – CIMMYT, Sustainable Intensification Program, House 10/B, Road 53, Gulshan-2, Dhaka 1213, Bangladesh; ‡ International Maize and Wheat Improvement Centre – CIMMYT, Sustainable Intensification Program. c/o Royal Tropical Institute, Mauritskade 63, Amsterdam 1092 AD, the Netherlands; § International Maize and Wheat Improvement Centre – CIMMYT, Sustainable Intensification Program. c/o ICRAF House, United Nations Avenue, Gigiri, Nairobi, Kenya; ¶ French Agricultural Research Centre for International Development – CIRAD, UPR AIDA, University of Montpellier. Avenue Agropolis, 34398 Montpellier Cedex 5, France; †† Department of Environmental Studies, University of California, 1156 High St, Santa Cruz, CA 95064, USA; ‡‡ International Maize and Wheat Improvement Centre – CIMMYT, Sustainable Intensification Program –Mexico, Apdo. Postal 6–641 06600, Mexico, D.F., Mexico

## Abstract

Intended to test broad hypotheses and arrive at unifying conclusions, meta-analysis is the process of extracting, assembling, and analyzing large quantities of data from multiple publications to increase statistical power and uncover explanatory patterns. This paper describes the ways in which meta-analysis has been applied to support claims and counter-claims regarding two topics widely debated in agricultural research, namely organic agriculture (OA) and conservation agriculture (CA). We describe the origins of debate for each topic and assess prominent meta-analyses considering data-selection criteria, research question framing, and the interpretation and extrapolation of meta-analytical results. Meta-analyses of OA and CA are also examined in the context of the political economy of development-oriented agricultural research. Does size matter? We suggest that it does, although somewhat ironically. While meta-analysis aims to pool all relevant studies and generate comprehensive databases from which broad insights can be drawn, our case studies suggest that the organization of many meta-analyses may affect the generalizability and usefulness of research results. The politicized nature of debates over OA and CA also appear to affect the divergent ways in which meta-analytical results may be interpreted and extrapolated in struggles over the legitimacy of both practices. Rather than resolving scientific contestation, these factors appear to contribute to the ongoing debate. Meta-analysis is nonetheless becoming increasingly popular with agricultural researchers attracted by the power for the statistical inference offered by large datasets. This paper consequently offers three suggestions for how scientists and readers of scientific literature can more carefully evaluate meta-analyses. First, the ways in which papers and data are collected should be critically assessed. Second, the justification of research questions, framing of farming systems, and the scales at which research results are extrapolated and discussed should be carefully evaluated. Third, when applied to strongly politicized topics situated in an arena of scientific debate, as is the case with OA and CA, more conservative interpretations of meta-analytical results that recognize the socially and politically embedded nature of agricultural research is are needed.

## INTRODUCTION

Farming looks mighty easy when your plow is a pencil and you’re a thousand miles from the corn field. – Dwight D. Eisenhower (1956)

Initially developed by medical researchers to synthesize data from multiple clinical trials, systematic literature review and meta-analysis are increasingly popular in the agricultural sciences. Systematic literature reviews apply a structured methodology to collect and analyse secondary data, with the objective of transparently reviewing all available research evidence (Borenstein et al., 2009). Systematic reviews contrast with traditional literature reviews, the former being thought of as more objective, defensible and conclusive (Borenstein et al., 2009; Garg *et al*., 2008; Gurevitch et al., 2018).

Meta-analysis takes systemic literature review further, fundamentally changing how research syntheses are conducted (Gurevitch *et al*., 2018). It extracts and assembles quantitative information from primary studies to build a database for analysis. This enables increased statistical power and the testing of hypotheses that can only be partially addressed through individual studies. Rosenthal and Schisterman (2010) suggest that meta-analysis permits researchers ‘…to formally and systematically pool together all relevant research in order to clarify findings and form conclusions based on all currently available information’ (p. 427). Most researchers conductingmeta-analysis collect means and standard deviations of response variables to determine treatment effect size (Hedges *et al*., 1999). Meta-analysis of combined data from papers that individually report non-significant or idiosyncratic relationships between variables can point to an underlying data structure across studies. Both Garg *et al*. (2008) and Borenstein *et al*., (2009) therefore argued that increased statistical power is a key reason for deploying meta-analysis to address conflicting research findings and resolve scientific debates.

**Figure 1:**
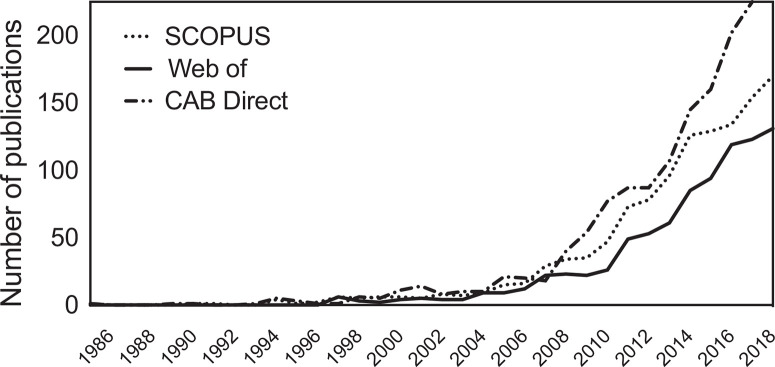
Annual growth in the number of papers recovered from thee bibliographic databases using ‘meta-analysis’ and ‘agricultur*’ in the title, keywords, or abstract (as of December 31, 2018).

Doré *et al*. (2011) recommended that agronomists conduct meta-analysis to investigate patterns in cropping system performance. Over 1000 studies using metaanalysis in agriculture have been published since 1985, with 65% completed since 2012 (Figure 1). Described as one of the most objective and robust methods in agricultural research (cf. Fisher, 2015), the usefulness of meta-analysis has however long been questioned in other fields. For example, Eysenck (1978) described metaanalyses of clinical psychotherapy interventions as ‘an exercise in mega-silliness’ and an ‘abandonment of scholarship’ because researchers commonly included studies ‘mostly of poor design’ (p. 517). Fitz-Gibbon (1984) and Eisler (1990) similarly critiqued early meta-analyses of educational and psychology studies. In a more recent evaluation of 9135 papers labelled as systematic review or meta-analysis in health care, Ioannidis (2016) found one in six studies to be misleading, and one in three redundant, unnecessary, or potentially biased.

Additional methodological concerns with meta-analysis have been identified in other fields that may be applied to the agricultural sciences. The first concern involves the criteria used to select and analyse literature. Failure to locate all available literature, or inclusion of primary studies with diverging or poorly implemented methods can lead to contradictory or erroneous conclusions (Englund *et al*., 1999; Garg *et al*., 2008; Haidich, 2010; Philibert *et al*., 2012). The greater availability of publications in developed compared to developing countries, and reduced accessibility of non-English literature (cf. Elsevier, 2009) may also compromise the comprehensiveness of research results. Publication bias, a condition resulting from journals’ preference to publish studies with significant rather than non-significant results, is one of several related issues (Garg *et al*., 2008; Gurevitch *et al*., 2018; Haidich, 2010; Murtaugh, 2002; Philibert *et al*., 2012). Analytical techniques are now available to overcome publication bias, though they are inconsistently applied (Gurevitch *et al*., 2018; Philibert *et al*., 2012).

Reviews of meta-analysis in agriculture include Philibert *et al*. (2012) and Brandt *et al*. (2013) who suggest that the methodological quality and application of meta-analytical techniques has been highly variable. Most meta-analyses in agronomy focus on crop yield response to experimental manipulation (Philibert *et al*., 2012). Yield is however only one criterion by which the performance of cropping systems can be judged: yield stability and resilience, nutritional yield and environmental and economic performance are additional relevant but less studied indicators.

Aside from the constructive critiiques of Philibert *et al*. (2012) and Brandt *et al*. (2013), critical appraisal of meta-analysis in the agricultural sciences is largely lacking. This paper addresses this research gap considering a suite of yet-unaddressed issues of importance, starting with the ways in which meta-analytical research is framed. Framing can be defined as the way in which research questions and methods are selected, described and justified as contributing to solutions for particular problems (Bardwell, 1991), for example, agricultural productivity or environmental goals. When applied to rural development, Andersson and Sumberg (2015) refer to studies that reiterate these goals as belonging to ‘development-oriented agronomy’. Given heightened competition among agricultural scientists for decreasing research funds, research topics and investments are commonly justified using the language of development-oriented agronomy (Andersson and Sumberg, 2015; Leeuwis *et al*., 2017).

Meta-analysis may be also described as a descendent of the logical-positivist tradition of science that champions empirical and hypothesis-driven inquiry as the prime mechanism by which unbiased knowledge is generated and validated. Sumberg *et al*. (2014) and de Roo *et al*. (2019) conversely recognized the sociopolitically embedded nature of agricultural science. By doing so, they recognize the ways in which agricultural researchers in development-oriented agronomy experience tension between the generation of scientific evidence and the need to convince multiple audiences (e.g., farmers, donors, other scientists and policy makers, among others) of the relevance of their research findings and types of agronomic practices. In addition to the narrative employed when agronomists design, interpret and discuss research results, we explore the ways in which this tension can influence the range of potential solutions to agricultural problems that may be proposed by agronomists conducting meta-analysis (Sumberg *et al*. (2014)).

Confirming the placement of meta-analysis within the logical positivist tradition, researchers publishing meta-analyses in agronomy frequently highlight the size and representativeness of their datasets – which are usually constructed using observations from small-plot agronomic experiments – to answer agricultural development questions of continental or even global significance (cf. Corbeels *et al*., 2014b; Knapp and van der Heijden, 2018; Lundy *et al*., 2015; Pittelkow *et al*., 2015a, 2015b). Goulding *et al*. (2011) however cautioned that results from small-plot trials should be interpreted cautiously, as they may not include higher level processes and contextual interactions, and thus poorly approximate whole field- and farm-scale performance.

Addressing these topics, we examine how meta-analysis has been used to support claims and counter claims over organic agriculture (OA) and conservation agriculture (CA). In doing so, we critically assess the suggestion that meta-analysis can provide unifying conclusions and rectify topics of scientific debate (cf.Bornstein *et al*., 2009 ; Fisher *et al*., 2015 ; Garg *et al*., 2008 ; Rosenthal and Schisterman, 2010). We adopt a ‘political agronomy’ perspective that recognizes the socio-politically embedded nature of agricultural science and suggests that agronomy can be an arena for contestation and debate (Sumberg *et al*., 2012). OA and CA are among the most widely disputed subjects in contemporary agronomy, with vigorous debate indicating large rifts in epistemological approaches and contrasting agricultural research and development paradigms (Sumberg *et al*., 2014). Considering these issues, we review prominent OA and CA meta-analyses published since 2007 and discuss whether meta-analysis has reduced or resolved research debate. We conclude by offering suggestions for how both scientists conducting meta-analyses, as well as the readers of scientific literature can more carefully evaluate meta-analytical evidence, particularly when applied in the context of development-oriented agronomy.

## METHODOLOGICAL APPROACH

This paper examines meta-analyses of OA and CA published since 2007 (Tables 1 and Table 2). Our study is not exhaustive but is focused on recent and prominent meta-analyses. Each case study considers primary data selection criteria and methodology following suggestions for cropping systems comparisons (cf. Cassman, 2007; Connor, 2013) and meta-analytical procedures in agronomy (Philibert *et al*., 2012). Our review also offers new insights by considering the ways in which authors rationalize and frame meta-analytical research questions, in addition to the justification given for, and consequences of, extrapolating results beyond the experimental setting. We also examine the discourse presented in case study metaanalyses by recognizing the political and socially embedded nature of agricultural research.

**Table 1:** Summary of four prominent systematic and meta-analytical reviews of organic agriculture (OA).

	Study					
Criteria	Badgley et al.(2007a)	de Ponti et al. (2012)	Seufert et al. (2012)	Ponisio et al.(2015)	Hossard et al.(2016)	Knapp and van der Heijden (2018)
Type of study	Synthetic review with extrapolation modelling	Meta-regression	Meta-analysis	Meta-analysis	Meta-analysis	Meta-analysis and meta-regression
Research question justification and framing	OA may be a sustainable agricultural alternative to a more intensified version of the Green Revolution to help feed the world. It is necessary to investigate the principle objections against organic agriculture making a significant contribution to global food supply.	Response to debate on the contribution of OA to the future of world agriculture, in which yield comparisons are central. Organic agriculture’s future role will largely be determined by economically competitive with organic agriculture, in which productivity plays a central role.	OA is often proposed as a solution for the major challenges of the global food system, including rising demand for food and the need to minimize environmental impacts. Critics however argue that lower organic yields will result in agricultural land expansion and environmental externalities.	Acknowledges the need to maintain or increase food production without sacrificing sustainability or resilience. Positions OA as the most widely practiced and studied ecological alternative to conventional agriculture, but acknowledges inadequacies and divergent research results and debate from existing studies, while seeking to resolve them.	OA and conventional agriculture are commonly framed as diametrically opposed production methods. There is a lack of evidence on the performance of low-external input systems as a third alternative to address food production and environmental sustainability.	Population growth increases global food demands. Production increases must be sustainable. Yield stability under organic systems is inadequately addressed.
Primary response variable	Yield	Yield	Yield	Yield	Yield	Yield and yield stability (relative stability ratio)
Crop(s)	Cereals, roots and tubers, sugars, legumes, tree, oilseed and horticultural crops, fruits, meat and dairy	Cereals, roots and tubers, oilseeds, horticultural crops, fruits, fodders.	Cereals, horticultural, oilseed, legume and fruit crops	Cereals, legumes, fruits and nuts, oilseeds, roots and tubers, horticultural crops	Maize, wheat	Cereals, legumes, fruits and nuts, oilseeds, roots and tubers, horticultural crops, tree crops
Paired comparisons (*n*)	293	362	316	1,071	66	443
Geography	North America (28%)Europe (27%)S. Asia (13%)Africa (9%)Latin America (8%)Asia (6%)S.E. Asia (6%)Middle East (1%)Caribbean (1%)Oceania (1%)	Europe (47%)North America (34%)Latin America (6%)Middle East (5%)S. Asia (4%)S.E. Asia (1%)Africa (1%)Asia (1%)	North America (52%)Europe (32%)S. Asia (8%)Africa (4%)Oceania (2%)Latin America (1%)Asia (1%)	Europe (46%)North America (45%)S. Asia (4%)Middle East (1)Latin America (1%)Africa (1%)Asia (1%)Oceania (1%)	North America (58%),Europe (42%)	North America (66%)Europe (20%)Asia (13%)Oceania (1%)
Comparison system (control)	Non-organic§	Conventional agriculture	Conventional agriculture	Conventional agriculture	Low external input or conventional agriculture	Conventional agriculture ¶
Replicable procedure*	Yes	Yes	Yes	Yes	Yes	Yes
References available*	Yes	Yes	Yes	Yes	Yes	Yes
Within-and between-study analysis*	No	No	Yes	Yes‡‡	Yes	Yes^k^
Sensitivity analysis*	No	No	Yes††	Yes	Yes	Yes
Publication bias assessed*	No	No	Yes	Yes	Yes	Yes
Data weighed*	No	No	Yes	Yes	Yes	Yes
Software described*	No	Yes	Yes	Yes	Yes	Yes
Dataset availability*	Yes	Yes	Yes‡‡	Yes	Yes	Yes
Clear definition of systems comparison†	No l	Yes**	Yes	Yes†††	Yes	Yes l
Quantifying and equalizing nutrient levels†	No	No	Yes§§	No	No, although variable rates are justified, described and discussed	No
Clarity in experimental design and replication†	No	Yes	Yes	Yes	Yes	Yes
Data sources‡	Grey literature (57%)Peer reviewed (36%)Conferences (7%)	Grey literature (65%)Peer reviewed (11%)Conferences (23%)	Peer reviewed (90.2%)Grey literature (9.2%)Conferences (0.3%)Theses (0.3%)	Peer reviewed (90%)Grey literature (10%	Peer reviewed (100%)	Peer reviewed (85%)Grey literature (15%)
Major conclusions	Organic agriculture can supply enough calories to feed the world at current and increasing *per capita* levels. The yield response ration is slightly *<*1.0 and *>*1.0 in the developed and developing world, respectively.	Organic yields ∼80% of conventional agriculture, but with large variation (SD 21%). Organicconventional yield gap increases as conventional yields increase.	Organic yields are lower than conventional systems, but the range depends on system and site characteristics, ranging from −5 to −34%.	Previous meta-analyses are flawed because of pseudo-replicationand Type-1 error. Following correction, organic yields were found to be 19.2% (±3.7%) lower than conventional. More diverse multi-crop and rotational systems decrease the yield gap.	Organic maize YRRs were *<* conventional. LEI yielded 1.25 times more than organic, and were not different than conventional. Pesticide and fertilizer N were lowered 53 and 36%. In winter wheat, LEI had lower YRRs than conventional, but were 1.43 times greater than organic, with 70 and 28% less pesticide and mineral N.	OA has significantly lower temporal yield stability (−15%) compared to conventional agriculture.

*, † Criteria identified summarized in Brouder and Gomez-Macpherson (2014).

‡ Grey literature includes academic and research sources lacking evidence of peer review. Conferences include those with edited published proceedings.

§ Includes comparisons from experiments and whole-farm observations.

¶ Includes experiments, whole-farm observations, and comparisons between years (before/after transition to organic or ‘agroecological’ management).

** Certified organic standards following IFOAM.

†† Includes sensitivity analysis of study quality, non-food rotation, experimental longevity, low versus high input, similar system components, best organic mgt., legumes and perennials, and best organic performance, with and without legumes.

‡‡ Includes dataset of studies rejected for meta-analysis through quality control.

§§ Supplementary information provides analysis of variable nitrogen inputs in organic and conventional, presenting effect size when N use was ‘similar’, compared under a variety of circumstances.

¶¶ ‘ Organic’, ‘ecological’ farm, production cropping and agricultural systems were searched for in studies with ‘compare’ and ‘yield’ as Boolean search terms.

*** Used random effects model with additional sources of variation considered, including between studies, within study between years, and response ratio differences within years, the latter signifying sowing date trials. Nested observations when taken from the same studies, to address independence assumptions, in response to non-nesting and problems of independence identified in Seufert et al. (2012).

††† Groups organic with ‘ecological’ or similar systems without clarifying criteria for definitions in the case of Ponisio et al. (2015), and organic versus non-organic, with organic referring to ‘agroecological’, ‘sustainable’ or ‘ecological’, with non-synthetic nutrient cycling processes, limited use of synthetic pesticides, and with a focus on soil quality regeneration, in the case of Badgley et al. (2007a), though subsequent critique pointed out that many observations included made use of synthetic fertilizers (cf. Avery, 2007).

**Table 2:** Summary of prominent systematic and meta-analytical reviews of conservation agriculture (CA).

	Study										
Criteria	Van den Putte et al. (2010)	Rusinamhodzi et al. (2011)	Zheng et al. (2014)	Corbeels et al. (2014b)	Pittelkow et al. (2015a)	Rusinamhodzi (2015)	Pittelkow et al. (2015b)	Lundy et al. (2015)	Huang et al. (2015)	Steward et al. (2017)	Knapp and van der Heijden (2018)
Type of study`	Meta regression	Meta-analysis	Meta-analysis	Meta-analysis	Meta-analysis	Meta-analysis	Meta-analysis	Meta-analysis	Meta-analysis	Meta-regression	Meta-analysis and meta-regression
Research question justification and framing	Soil degradation and erosion are important problems. CA is as a proposed solution, but systematic yield assessments are limited.	Soil degradation and erosion are important problems in smallholder farming systems. CA is as a proposed solution, but systematic yield assessments are limited.	CA is a recommended practice for sustainable crop production, but yield variability has been inadequately assessed	Soil degradation and erosion are important problems in smallholder farming systems. CA is as a proposed solution, but systematic assessment of yield stability is lacking.	CA is proposed as a method to address growing food security and development challenges, though impacts of no-tillage on yield remain contested.	Crop rotation and residue retention are important for yield and yield stability, but inadequately studied under CA.	NT may be important for feeding a growing world population while providing environmental and economic benefits, though impacts of no-tillage on yield remain contested.	CA is actively promoted in Africa and described as erosion controlling and ‘climate smart’, though yield outcomes may be limited by farmers’ ability to manage soil fertility.	Rice is critical for food security. Laborsaving and soil conserving technologies are needed. Environment and management effects on NT yield are poorly understood.	Climate change threatens food security in Africa. CA may be a ‘climate smart’ management option, but yields under different stresses, soils, and management practices have not been systematically assessed.	Population growth increases global food demands. Production increases must be sustainable. Yield stability under CA is inadequately addressed.
Primary response variable	Yield	Yield and yield stability	Yield	Yield and yield stability	Yield and yield stability	Yield	Yield and yield stability	Yield and yield stability	Yield and yield stability	Yield and yield stability as a function of precipitation and heat stress	Yield and yield stability (relative stability ratio)
Crop(s)	Fodder maize, grain maize, potato, sugar beet, spring and winter wheat	Maize	Cereals	Cereals, legumes, cotton	Multiple cereals, legumes, roots and tubers, tree crops, vegetables	Maize	Multiple cereals, oilseeds, legumes, roots and tubers, tree crops, vegetables	Cereals, legumes, roots and tubers, tree crops, vegetables	Rice	Maize	Multiple cereals, oilseeds, legumes, roots and tubers, vegetables
Paired comparisons (*n*)	563	364	123	261	5463	688	6005	2759	265	1042	2453
Geography	Europe (100%)	North America (50%) Africa (19%) Latin America (12%) S. Asia (*<*0.1%) Asia (*<*0.1%) Europe (*<*0.1%) Oceania (*<*0.1%)††	Asia (100% China)	Africa (100%)	North America (57%) Europe (12%) Africa (6%) Latin America (6%) S. Asia (9%) Asia (4%) Oceania (4%) Middle East (2%)	North America (33%) Africa (50%) Latin America (5%) S. Asia (5%) Asia (2%), Europe (2% Oceania (2%)	North America (38%) Europe (17%) Africa (9%) Latin America (8%) S. Asia (9%) Asia (8%) Oceania (8%) Middle East (3%)	North America (46%) S. Asia (18%) Europe (12%) Africa (8%) Latin America (6%) Asia (6%) Oceania (3%) Middle East (1%)	Asia (100%, all China)	Africa (72%) North America (16%) Latin America (6%) Asia (5%) Oceania (1%)	North America (60%) Asia (15%) Europe (10%) Oceania (7%) Africa (4%) Latin America (3%) Middle East (2%)
CA practice(s)	NT and RT	RT, NT, NT + rotation, NT + rotation +residues	CT, NT. And RT without residues, NT with residues	NT, NT + residues, NT + residues +rotation	NT + residues, NT +residues +rotation	NT, NT + rotation, NT +rotation +residues	NT	NT with residues OR without residues	NT	NT + residues, NT +residues +rotation	NT + residues, NT+ residues + rotation
Comparison system (control)	CT (residue management unspecified)	CT (residue removed)	CT residues removed	CT residues removed	CT with residue incorporated	CT	CT with residue incorporated	CT with residues OR without residues (paired with NT)	CT	CT with residue incorporated, burnt, or removed	CT with residue incorporated
Replicable procedure*	Yes§	Yes**	Yes	Yes	Yes	Yes**	Yes	Yes	Yes	Yes	Yes
References available*	Yes	Yes	Yes	Yes	Yes	Yes	Yes	Yes	Yes	Yes	Yes
Within- and between study analysis*	No	Yes	Yes	No	Yes	No	Yes	Yes	No	No	Yes
Sensitivity analysis*	No	No	No	No	No	No	Yes	Yes	No	No	Yes
Publication bias assessment*	No	No	No	No	No	No	Yes	Yes	Yes	No	Yes
Data weighted*	No	Yes	Yes	Yes	Yes	Yes	Yes	Yes	Yes	Yes‡‡	Yes
Software described*	Yes	No	Yes	Yes	Yes	No	Yes	Yes	Yes	Yes	Yes
Dataset availability*	No	No	Yes	Yes	Yes	No	Yes	No	Yes	Yes	Yes
Clear definition of systems comparison†	¶	Yes	No	Yes	Yes	Yes	Yes	Yes	Yes	Yes	Yes
Quantifying and equalizing nutrients†	Unclear	Yes	Yes	Yes	Yes	Yes	Yes	Yes	Yes	Yes	Yes
Clarity in experiment design and replication†	Yes	Yes	No	Yes	Yes	Yes	Yes	Yes	Yes	No	Yes
Data sources	Peer reviewed (70%) Conference proceedings (30%)	Peer reviewed (88%) Grey literature (12%)	Peer reviewed (100%)	Peer reviewed (100%)	Peer reviewed (100%)	Peer reviewed (90%), conference proceedings (2%), theses (7%)	Peer reviewed (100%)	Peer reviewed (100%)	Peer reviewed (100%)	Peer reviewed (100%)	Peer reviewed (100%)
Major findings	NT reduces yield 8.5%. Strategic deep tillage and diversified rotations reduce negative effects.	Crop rotation with high N is crucial for CA. Mulch cover in high rainfall areas lowers yields by waterlogging. CA needs to be targeted and adapted.	Differential CA effects result from regional variation climate and crops. CA increases maize but reduces wheat yield. Residues are needed for maize and seasons with warm/dry climates.	NT without residues or rotation depresses yield. CA responds best to high N rates. Precipitation had no effect because most studies failed to report within season rainfall.	NT reduces yields 5.7% overall. Residue retention and rotations mitigate this effect, but not entirely.	CA yield advantages only significant with high N rates and low precipitation.	NT yields are reduced without N addition. Site-specific adaptation of NT systems is needed to attain yield goals.	Nitrogen fertilization is important in counteracting yield declines in NT systems.	NT decreased yield in rice-rice but increased in rice-upland systems, though with variation depending on climate and soils.	CA improves maize yield with increasing drought or heat stress with interaction between soil moisture and heat stress mediated by soil clay content.	Temporal yield stability under NT does not differ significantly from CT; transition to NT does not affect yield stability

CA refers to conservation agriculture. NT (without residue or rotation, unless specified), RT and CT indicate no-, reduced- and conventional-till, respectively. YRR indicates yield response ratio.

*,† Summarized in Table 3.

‡ Grey literature includes academic and research sources lacking evidence of peer review. Conferences include those with edited published proceedings.

§ Statistical analysis replicable, literature search not replicable due to lack of clear description on search terms and databases utilized.

¶ Criteria for what qualifies as reduced tillage not clarified, with the exception of lack of soil inversion. Unclear if no-tillage treatments involve residue retention or rotation.

** Journal databases used not clarified.

†† Percentages indicate study number rather than paired observations.

‡‡ Observations weighted by replication, plot and yield sampling area.

## CASE STUDY DESCRIPTIONS

### Organic agriculture

OA is defined by the International Federation of Organic Agricultural Movements (IFOAM) as a production system that sustains the health of ecosystems and people, and that makes use of ecological processes and cycles to eliminate synthetic inputs (IFOAM, 2015). OA is frequently equated with ‘ecological’, ‘agroecological’, ‘sustainable’ and/or ‘low-external input (LEI)’ agriculture, though each may differ in practice (Magdoff, 2007; Rigby and Caceres, 2001; Shennan *et al*., 2017). OA is also generally contrasted with ‘conventional agriculture’, although the characteristics of conventional agriculture tend to be counterfactually defined as anything not organic (Giller *et al*., 2017: 154). Conversely, OA is commonly framed as a holistic and sustainable alternative production system, as well as a philosophy (Rigby and Caceres, 2001).

Although debate over OA has a long history and has been recognized as being rooted in schisms between different agricultural paradigms (Beus and Dunlap, 2010), a systematic review by Badgley *et al*. (2007a) concluding that OA could produce more food than required to feed the global population sparked much contemporary debate and paved the way for use of meta-analysis in OA-conventional systems comparisons. Subsequent and consecutive meta-analyses examining OA each claimed increasingly large datasets and comprehensive and conclusive analyses (de Ponti *et al*., 2012; Ponisio *et al*., 2015; Seufert *et al*., 2012). This case study analyses key meta-analyses published following Badgley *et al*. (2007a) and considers if meta-analysis has resolved or contributed to further debate over the merits of OA.

### Conservation agriculture

CA involves three crop management principles. These include minimal soil disturbance (reduced or no tillage (NT)), crop residue retention as mulch and crop rotation or diversification. Practiced in combination, these principles are meant to reduce soil degradation while increasing yields and reducing production costs (FAO, 2017).

Although reduced tillage (RT) dates to the 1930s, widespread adoption began only after 1970, following the release of herbicides, mechanized NT planters and, in the 1990s, the advent of herbicide resistant, genetically modified crops (Giller et al., 2015). Erosion mitigation and reduced costs from the elimination of tillage appear to have been major drivers of adoption on large-scale farms in developed countries. These goals were however also considered imperative for smallholders in developing nations (Ekboir, 2001), sparking interest amongst international research and development organizations in CA (Giller et al., 2009).

CA has since been widely reframed as a yield-enhancing technology to improve smallholder food security, with widespread promotion to smallholder farmers ensuing in sub-Saharan Africa and South Asia, in particular (Andersson and D’Souza, 2014; Giller et al., 2009, 2015). This prompted critical debate over the suitability of CA in the context of development-oriented agronomy, and particularly the yield and adoption claims made for CA (Giller et al., 2009). This case study consequently considers eleven prominent meta-analyses on CA published since 2010, again asking if meta-analysis has resolved or inadvertently contributed to further debate.

## CASE STUDIES

### Meta-analysis and organic agriculture

Contemporary debate over the productivity of OA emerged with Badgley et al. (2007a), who framed their paper as a response to objections that OA could make significant contributions to the global food supply. Badgley et al. (2007a) compiled what they referred to as a ‘global dataset’ (p. 86) of 239 yield response ratios (YRR, the ratio of organic to conventional yields,) for a diversity of crop, meat, and dairy products (Table 1). An average YRR of 1.32 was reported, indicating higher organic than conventional yields, with ratios in developing and developed countries averaging 1.80 and 0.92, respectively. Average ratios were extrapolated to estimate if OA could produce sufficient calories to meet global requirements. The authors concluded that OA could supply 17–50% more calories person–1 than the globally extrapolated average adult requirement per day. Badgley et al. (2007a) also summarized 77 studies quantifying biological nitrogen fixation to estimate if legumes could supply sufficient of nitrogen annually to substitute for global use of synthetic fertilizer N. They concluded that OA could supply global food requirements without requiring additional land or fertilizer resources, and advocated strongly for increased institutional and public support for OA.

The editors of Renewable Agriculture and Food Systems, which published the study, also permitted Badgley et al. to publicly reply to Editor and peer-reviewers’ concerns with their manuscript in a special Forum. Agronomists presented a range of technical critiques used to problematize Badgley et al.’s results and argue against increased OA research funding and support. Cassman (2007), for example, critiqued the analysis of YRRs from singly grown crops, as opposed to rotational systems commonly employed in OA. Use of grey literature and concern over yield data collected in different years, e.g. before and after farmers adopted organic practices, were flagged as methodologically invalid. Such before–after measurements comprised half of the data from developing countries presented by Badgley et al. (2007a), and originated from a single report (cf. Pretty and Hine, 2001). Badgley and Perfecto (2007) however countered that organic-conventional comparisons were rare in developing countries, necessitating the use of before–after comparisons and grey literature.

Importantly, Badgley et al.’s framing of OA was broad, including agroecological, sustainable or ecological practices that either exclude or make limited use of synthetic pesticides, and that improve soil quality. This definition differs from IFOAM and other certifying agencies, and was critiqued by Cassman (2007) as vague. The food policy analyst Dennis Avery argued that nearly half the studies in the Badgley et al. (2007a) database used synthetic fertilizer or pest control products (Avery, 2007), which would disqualify them as organic under most certification programmes. Badgley et al. (2007b) countered that practices using synthetic inputs in ways intended to reduce their application should still qualify as OA.

Cassman (2007) and Connor (2008b) also suggested that high YRRs were an artefact of contrasting intensified organic management with resource-constrained subsistence agriculture in developing nations. Further critique focused on the application of unbalanced nutrient application rates where organic systems receive organic manure but conventional crops do not, and on the need to quantify the effect of caloric yield per unit area and time in organic rotational systems with cover crops, rather than yield per hectare in a single season.

Five key meta-analyses were subsequently published. The first framed their analysis in response to the emerging debate and as an assessment of the role of OA in the future of world agriculture. de Ponti et al. (2012) utilized 362 organic-conventional yield comparisons, collected exclusively from peer-reviewed sources in which OA treatments met IFOAM standards (Table 1). They therefore rejected 86% of the data presented by Badgley et al. (2007a), and concluded that OA yields are on average 20% lower than yields under conventional management, but with large variance. Exponential regression showed that the gap between organic and conventional yields grows as conventional yields increase and OA becomes nutrient limited, addressing earlier critique that OA should be compared with conventional best management practices rather than subsistence systems (cf. Cassman, 2007; Connor, 2008b).

Seufert et al. (2012) framed their meta-analysis in response to the proposition that OA can be a solution to major challenges in the global food system, including the need to minimize environmental impacts and agricultural land expansion from low-yielding farming systems. Sixty-six primary studies, 90% peer-reviewed, were used to generate 316 yield comparisons in which OA conformed to commercial organic certification standards (Table 1). Only primary studies reporting means and variance were included, with cumulative effect size weighted by individual or multi year observations of variance in the hierarchical, categorical mixed model employed. Seufert et al.’s results indicated 5 to 34% lower organic yields, depending on cropping system and site characteristics. They however cautioned that yield is but one of many metrics by which OA should be judged, while also suggesting that ideologically charged debate should be minimized in favour of systematic evaluation. Nature also published a Forum on Seufert et al. (2012), in which Reganold (2012) positively interpreted their meta-analysis as evidence that can ‘…underscore the potential for organic farming to have an increasing role in a sustainable food supply’ (p. 176), while Dobermann (2012) considered the problematic nature of field-scale experimentation and suggested that ‘ It is time to accept that various types of agriculture can have a place in feeding the world, depending on the availability of land, the degree of self-reliance of agricultural systems in terms of critical inputs to value chains (such as nutrients and other resources), the scale of food production, and the desired and feasible trade in agricultural goods’ (p. 177). Connor (2013) conversely argued that Seufert et al. (2012) were misguided and lacked critical thinking that ‘…adds confusion to the current debate of which food production systems can best feed and green a world expected to reach 9.2 billion human inhabitants by 2050’, while proposing that ‘The solution must be found in greater yields and cropping intensity’ (p.146).

Framing OA as the most feasible ecological option that responds to the imperative to ‘adopt resilient and sustainable agricultural practices as soon as possible’, Ponisio et al. (2015:1) aimed to provide new evidence and argued that all previous organic conventional comparisons were methodologically flawed. They specifically critiqued Badgley et al. (2007a) for not accounting for variance or applying probability statistics. While Seufert et al. (2012) did account for variance, Ponisio et al. (2015) nonetheless critiqued the study for combining nested data from multiple trials without accounting for hierarchy in ways that introduce pseudo-replication and the risk of type-1 statistical error. Ponisio et al. (2015) therefore assembled an ‘…extensive dataset including over three times more yield comparisons than previous studies’ (p. 4) represented by 1071 comparisons from 115 studies contrasting conventional from organic and ecological agriculture (Table 2). A hierarchical regression model was used to account for between- and within-study random variation, as well as between- and within-year variation, with random effects nested within studies. Ponisio et al. (2015) found average organic and ecological yields to be 19% lower than conventional systems. Where diversified crop rotations and multi-cropping were practiced, this yield gap was declined to 9±4% and 8±5%. They concluded that investment in analytically rigorous research aimed at eliminating this yield gap is justified given the urgent need for more sustainable and resilient production systems that overcome improve livelihoods of the rural poor.

Responding to these debates and recognizing the lack of information on LEI agriculture as an alternative to both conventional and OA, Hossard et al. (2016) provided a more nuanced analysis. Their study analysed LEI maize and wheat systems in the US and Europe. While organic maize yields were 1.71 Mt ha–1 lower than conventional yields, LEI had a YRR averaging 1.25 times more than OA, while being statistically indistinguishable from conventional agriculture. Pesticide and fertilizer N use was reduced by 50 and 36%, respectively, in LEI compared to conventional systems. Organic winter wheat yields were also lower than with conventional practices, but with 70 and 28% less pesticide and mineral N inputs. LEI winter wheat conversely yielded 1.43 times more than OA.

Framing their work as a comprehensive response to the promotion of OA an ‘environmentally friendly’ method proposed to meet population growth and food security challenges, Knapp and van der Heijden (2018:1) presented what they termed as a ‘global meta-analysis’ of yield stability over time in both OA and CA compared to conventional agriculture. Utilizing data provided by Ponisio et al. (2015), they analysed 443 multiple-year observations, 86% of which were derived from studies in developed nations (Table 1). Considering OA, they concluded that OA has 15% lower temporal yield stability than conventional systems, and suggested increased emphasis on studies of the resilience of cropping systems considering growing population and food demands.

### Meta-analysis and conservation agriculture

Framed considering the need to arrest soil degradation in different environments, Van den Putte et al. (2010) provided the first meta-analysis described as an evaluation of CA by assembling 563 European comparisons for five crops. They concluded that RT without crop residue retention leads to significant yield reductions of 13 and 4% in maize and winter cereals, respectively. NT with residues retained resulted in a 8.5% reduction in yield relative to conventional tillage, although residue management practices under the latter were not clearly defined. RT with surface residues conversely reduced yields by ca. 4.5%. Van den Putte et al. (2010) also unpacked environmental and management influences on NT and RT performance, concluding that biotic stresses and deep seed placement reduces yields in dry climates/years, but not on sandy or clayey soils. Rusinamhodzi et al. (2011) framed their paper in light of soil degradation concerns, while adding emphasis to smallholder farming systems. They used 364 paired comparisons to identify how long-term mulch retention, rotation, effects and precipitation regimes influence maize yield responses to CA, compared with conventional tillage under sub-humid climates. Their results, largely based on data from the Americas and sub-Saharan Africa, indicated increasing CA maize productivity over time when residues were retained with rotations and high N inputs. NT or RT without residue retention however resulted in yield depression. Van den Putte et al. (2010) and Rusinamhodzi et al. (2011) therefore both suggested targeting and adapting CA to specific biophysical environments where these practices are most appropriate.

Zengh et al. (2014) open their paper indicating the need to resolve uncertainties limiting the ‘smooth and wide application’ of CA in China. Their meta-analysis of 123 comparisons characterized environmental influences on yield response in 5+ year duration maize, rice and wheat trials. NT was found to increase yield by 6.3% when residues were retained, particularly in environments with low or temporally unstable precipitation, though crop responses differed, and rotational effects were not considered. Corbeels et al. (2014b) conversely focused their meta-analysis in sub-Saharan Africa to study yield patterns with differing rotation, mulch and N rates. Their work was framed as an assessment of proposals that CA can limit soil degradation in smallholder farming systems and confirmed the importance of N fertilization, mulch and crop rotation to avoid yield depression. They found that NT without residues or rotation depresses yield, and that CA treatments responded best to high nitrogen rates.

Although the suitability of CA for smallholder farmers in developing countries had been questioned for several years (e.g. Giller et al., 2009), it was not until a study in Nature by Pittelkow et al. (2015a) that debate over the relevance of meta-analysis surfaced. Pittelkow et al. (2015a) describe their work as a ‘global meta-analysis’ (p. 365), which included 5463 yield comparisons from 43 crops across 63 countries with a robust methodological approach. Measured across all data, they concluded that NT lowers yields by an average of 5.7% relative to tillage for a variety of crops, although positive effects were found in more arid climates when rotations and residue retention were applied. Across climates and observations, the addition of rotations and residue retention to NT also reduce yield loss by 2.5%.

In comments sent to Nature, So et al. (2015) critiqued Pittelkow et al. (2015a) as geographically biased – and hence not globally representative – because 69% of observed datapoints were from North America and Europe alone. Others emphasized that measurements of yield under CA may not be as important as yield stability and resilience over time, while also suggesting that CA has crucial environmental benefits (Friedrich et al., 2015; So et al., 2015)^1^. Friedrich et al. (2015) criticized Pittelkow et al. (2015a) for insufficient details regarding how NT plots were managed in primary studies, leading to misclassification of CA treatments, a topic for which earlier guidelines had been proposed by Derpsch et al. (2014). Friedrich et al. (2015) did not however attempt to quantify the number of studies which may have been misclassified using the public database provided by Pittelkow et al. (2015a).

Khun and Hu (2015) conversely commented in Nature’s online comments that ‘…increased yields in drylands with conservation tillage have been acknowledged by Pittelkow et al. (2015a), but not fully appreciated with regard to food security. The benefits of CA on crop yields is of particular significance during dry years when famine in drylands is not caused by lack of global (average) production, but regional access to affordable food after poor harvests’. They concluded that ‘… in the light of the actual spatial and temporal dimensions of conservation tillage impacts on crop yields, conclusions drawn from oversimplifying meta-studies like that of Pittelkow et al. (2015a), based on one global average, carry the serious risk of contributing to poorly researched policy development and agricultural practice’.

Rusinamhodzi (2015) addressed these issues through meta-analysis of a smaller but similarly international dataset, though limited to maize and examining variants of CA practices (Table 2). He framed this study in light of how yield stability is inadequately studied under CA, and provided evidence that mulch has greater benefits in semi-arid environments. Yield benefits from CA also were found only when maize was rotated with legumes in arid climates and with N addition. Rusinamhodzi (2015) however positioned their results in a wider context by concluding that the maintenance of permanent soil cover is a challenge in mixed crop-livestock systems where trade-offs with feed are common. They therefore suggested targeting CA by socio-ecological niche.

Making use of the database initially developed by Pittelkow et al. (2014, 2015) and Lundy et al. (2015) analyzed 6005 and 2779 comparisons, respectively. The former framed their work in light of the potential environmental and economic advantages of NT in feeding a growing world population, while also carefully differentiating NT from CA in the papers’ supplementary materials. The latter strongly framed their work as informing debates around the appropriateness of CA in sub-Saharan Africa, though only 8% of their dataset included observations from this region. Each paper also heavily discussed CA, although analyses did not include CA sensu stricto. Rather, both papers concentrated on crop species, N management, and environmentally specific yield responses to NT, with or without crop residues, compared with conventional tillage with similar residue management (Table 2). Huang et al. (2015) also analysed 265 comparisons of the effect of NT on rice yield patterns in China, although they avoided discussion of CA entirely, despite considering residue retention and rotation in a portion of their dataset.

Framing their paper in terms of the importance of overcoming yield gaps, climate change and climate-smart agriculture in sub-Saharan Africa, Steward et al. (2018) applied meta-regression to 1042 CA (NT + residues, NT + residues + rotation) and conventional tillage (CT with residue incorporated, burnt or removed) comparisons for maize in moisture and heat stressed environments. Their analysis incorporated 42 studies and is unique because data were supplemented with weather observations used to quantify moisture and temperature stress. Results from the general linear mixed effects model employed indicated that CA yields improve with increasing moisture and heat stress although these effects are partially controlled by soil texture. Previous studies also suggested that N fertilization can offset lower CA yields (Corbeels et al., 2014b; Lundy et al., 2015; Rusinamhodzi, 2015; Rusinamhodzi et al., 2011; Pittelkow et al., 2015b). Steward et al. (2018) conversely provided new evidence that increasing N rates in CA does not improve maize yield under drought. And while previous metaanalyses suggested that rotation improves CA yield (Corbeels et al., 2014b; Pittelkow et al., 2015a; Rusinamhodzi, 2015; Rusinamhodzi et al., 2011; Van den Putte et al., 2010), Steward et al. (2018) found little supporting evidence, although diverse rotations were found to reduce yield variability under heat stress.

Last, Knapp and van der Heijden (2018) also framed their paper as a ‘global meta-analysis” in terms of the need to match growing population and food demand with sustainable productivity increases. They highlighted that temporal yield stability under CA remains poorly understood. A total of 2453 comparisons from trials at least 4 years in length were made by re-analysing data extracted from Pittelkow et al. (2015a), 60% of which came from North America. Absolute and relative stability YRRs were calculated as the ratio of experimental to treatment standard deviations or coefficients of variation across observational years, respectively. They found that temporal yield stability under NT does not differ significantly from conventional tillage, and that the transition to NT does not affect yield stability. Knapp and van der Heijden (2018) also discussed the limitations of experimental plot-scale measurements of yield stability relative to farm-scale measurements with multiple crops and crop rotations. In order to improve yield stability at this scale, they suggested ways that farmers could cultivate different crops in different fields to overcome poor performance of particular species in particular fields. They also suggested that use of species and genotype mixtures to reduce risks of crop failure.

## DISCUSSION

By applying powerful statistical analyses to large datasets constructed using primary literature, meta-analysis is intended to arrive at unifying conclusions and provide clarity in research (Borenstein et al., 2009; Fisher, 2015; Garg et al., 2008; Gurevitch et al., 2018). Use of meta-analysis may also be described as part of the logical-positivist paradigm, in which researchers justify and frame their work in terms of the primacy of hypothesis-driven and empirical inquiry. However, researchers’ paradigms can also be influenced by their politicized worldviews, beliefs, and perceptions of reality, in turn affecting scientific framing (Žukauskas et al., 2018). Our review, which recognizes the socially embedded nature of agricultural research (Sumberg et al., 2014), suggests that the variable application of methods and contestation over the framing and justifications given for research questions can undermine the purpose of meta-analysis to provide definitive conclusions.

Do the large sizes of databases and reportedly comprehensive analyses conducted with meta-analyses matter? Our case studies of OA and CA meta-analyses indicate that meta-analysis appears to fuel rather than diminish controversy. This is particularly the case for meta-analyses framed as contributing evidence to discourse that productivity increases are requisite for feeding a global population and assuring food security in smallholder agriculture^2^. We review these issues by discussing three considerations for how both scientists conducting meta-analysis and readers of scientific literature can more carefully evaluate meta-analytical evidence, particularly when applied in the context of development-oriented agronomy.

### Defining cropping systems and literature inclusion criteria

The field of science and technology studies has long acknowledged the problematic but necessary role of experiments in advancing knowledge. Experiments are a social construct intended as a simplified version of reality (Gooding et al., 1989). When designing cropping systems trials, agronomists make choices regarding the grouping and organization of a range of component crop management practices (e.g. tillage, irrigation, fertilization, etc.) into standardized categories (e.g. OA or CA) that can be mechanistically implemented across replicates. Yet as shown in our case studies, scientists encounter tension between the generation of experimental evidence and the need to justify their studies in terms of research investment and/or development relevance, and (in some cases) development impact (de Roo et al., 2019; Leeuwis et al., 2017; Sumberg et al., 2012).

Our review also highlights an additional weakness of meta-analysis when applied to agronomy. While experimental standardization permits replication and statistical inference, this process can actually decouple chosen management practices from the contextual realities of the farming systems are meant to represent. This limits the degree to which agronomists can responsibly extrapolate and discuss the implications of field trial results. Meta-analyses in agronomy appear to amplify this problem. Researchers conducting meta-analyses make additional choices regarding what treatment combinations and experimental procedures in primary studies they consider appropriate and admissible to their databases. Yet when databases are built on treatments that are debatably inappropriate, or that are highly decontextualized representations of farming systems realities, researchers extrapolating their results may inadvertently reduce the value of their studies to provide relevant and unifying conclusions.

The OA case study particularly highlights how differing paradigm and opinions regarding what does or does not constitute an appropriate treatment may render debates difficult to resolve, regardless of statistical power accrued by using metaanalysis. While screening literature for their systematic review, Badgley et al. (2007a) for example grouped ‘…farming practices that may be called agroecological, sustainable, or ecological; utilize natural (non-synthetic) nutrient-cycling processes; exclude or rarely use synthetic pesticides; and sustain or regenerate soil quality… [and] include non-certified organic’ (p. 87) as ‘organic’ in their analysis. This definition, which is arguably broader than most organic certification standards, resulted in a number of studies in which synthetic fertilizer had been applied being counted as OA. This broad definition resulted in considerable contestation (Avery, 2007; Cassman, 2007; Connor, 2008a). Subsequent OA meta-analyses by Seufert et al. (2012) and de Ponti et al. (2012) therefore applied formal organic product certification standards as the baseline criteria for literature and data selection.

Ponisio et al. (2015:1) later strongly framed their analysis in terms of the ‘imperative that we adopt sustainable and resilient agricultural practices as soon as possible’. They equated OA with generally better performance than conventional practices when sustainability indicators were considered, and therefore justified their research as an investigation into how crop diversification affects OA performance. Primary data were therefore collected from databases using Boolean searches for the terms ‘organic’ and ‘ecological’ with ‘agriculture’, ‘production’, ‘cropping’ and ‘yield’, as well as ‘compare’. They however did not provide clear definition for the specific management practices that constituted ‘ecological’ practices. This distinction is important because ‘ecological agriculture’ is generally broader than OA, and may make strategic and targeted use of synthetic inputs and may or may not conform to organic standards (Magdoff, 2007; Shennan et al., 2017). With the exception of Van den Putte et al. (2010), whose definition of what constitutes reduced and conventional tillage was not fully specified, the criteria used to define different configurations of CA principles in the meta-analyses reviewed in this paper tended to be more specific (Table 2). Such differences highlight that meta-analyses in agronomy are socially and politically situated, and that this may affect the definition of cropping system comparisons and literature selection.

To address these problems, Cassman (2007) proposed that cropping systems comparisons should only be considered where ‘best management practices’ are clearly specified and employed for each system studied. Yet defining what constitutes ‘best management’ is already a contested issue, and may be complicated by differing values and agricultural research paradigms (cf. Beus and Dunlap, 2010). Indicators of ‘best management’ and cropping systems performance (e.g. yield, profitability, efficiency, environmental or sociocultural outcomes) may also trade-off with each other. Selection of appropriate criteria for ‘best management’ practices therefore entails a degree of subjectivity. Questions of disciplinary authority and legitimacy may also arise over who is qualified to determine what constitutes ‘best management practices’. This appears to be particularly relevant in debates where development-oriented agronomy plays a role in research framing and justification, including OA and CA, among other topics such as the System of Rice Intensification (Andersson and Sumberg, 2015).

An example of these problems is provided by the CA case study. A widely recognized advantage of RT is to forgo time- and energy-consuming repetitive plowing. Tillage can also delay sowing and crop establishment, which may lower yield potential (Hobbs et al., 2008). Under these circumstances, RT and early sowing could be considered a ‘best management practice’. Yet in order to improve comparability of treatments (and increase the number of studies included in their database), more than half of the meta-analyses reviewed in this paper included data from studies in which CA and conventional treatments were established on the same date (cf. Knapp and van der Heijden, 2018; Lundy et al., 2015; Pittelkow et al., 2015a, 2015b; Steward et al., 2017). This observation underscores how the choices that scientists make when designing experiments and meta-analyses to isolate treatment effects may result in a decoupling from farmer realities. Keil et al. (2015), for example, argued that in the context of eastern India, Pittelkow et al.’s (2015a) conclusion that NT reduces wheat yield is invalid because farmers often utilize NT practices to advance wheat sowing dates. In addition to production cost reductions, this permits the crop to escape from yield-reducing late-season heat stress. Keil et al. (2015) illustrated this point with farm survey data, backing earlier observations by Erenstein and Laxmi (2008) in north eastern India where NT has been adopted by farmers on over 1.5 million ha, resulting in an estimated 5–7% increase in wheat yields.

These observations – which appear to be amplified in meta-analyses of OA and CA that use large datasets and claim increasingly comprehensive research results –represent the inherent tension in empirical studies that arises when scientists define treatments and interpret the implications of their results outside the experimental setting (Gooding et al., 1989). A first step in addressing this issue is to formally recognize that cropping systems may not be as simple in reality as compared to experimental settings. In other words, crop management follows a wide and variable range of practices when implemented by farmers as compared to experimental agronomists (Goulding et al., 2011; Shennan et al., 2017). Rather than relying on perhaps artificial groupings and diametrically opposed comparisons of OA or CA versus conventional management, research could also consider the importance of gradients in crop management. A preliminary example is the meta-analysis conducted by Hossard et al. (2016) that compared a range of LEI systems to organic and conventional agriculture. Another perhaps more important and general suggestion is to more conservatively interpret and extrapolate the implications of experimental and meta-analytical research results.

### Research question framing and the boundaries of meta-analysis

As described previously, the ways in which cropping systems are defined and studies are screened for meta-analysis can influence the interpretation of research results. The ways in which scientists conceive of and frame their research questions is of similar significance. Conceptual frameworks are important in the organization of human experience, perception, and understanding, including scientific paradigm (Žukauskas et al., 2018). The boundaries imposed by a conceptual framework help to define what information may or may not be considered valid in the evaluation of research evidence to inform decision making (Goffman, 1974). Most of the meta-analytical studies of OA and CA reviewed in this paper justify their work as contributing to development-oriented agronomic goals, e.g., meeting global food production, food security and environmental sustainability goals. In comparison to localized and context-specific research results, research that is framed as answering questions of broad global significance can arguably increase scientists’ chances of publication and citation. Where h-indices are highly rewarded in evaluating a researchers’ accomplishments, concerns have also emerged that meta-analysis can distort scientific integrity (Cohnstaedt and Poland, 2017; Gurevitch et al., 2018; Longo and Drazen, 2016). Leeuwis et al. (2017) and Andersson and Sumberg (2015) also point out that framing research in terms of global development impact is important for securing funding. Scientists discussing the implications meta-analytical results beyond the plot scale however face a number of important challenges.

For example, Corbeels et al. (2014b) and Rusinamhodzi (2015) both examined maize yields under CA in sub-Saharan Africa. Both studies are clear examples of meta-analysis framed within the political economy of development-funded agricultural research (Andersson and Sumberg, 2015). Having a clear and practical development orientation – where and by which farmers can yields can be increased through CA? – Corbeels et al. (2014b) and Rusinamhodzi et al. (2015) compared NT and treatments inclusive of all three CA principles to conventional tillage practices in which residues were removed from fields. Lundy et al. (2015) and Pittelkow et al. (2015a) conversely reframed the debate that had emerged over the benefits of CA in sub-Saharan Africa (cf. Giller et al., 2009) in terms of meeting global food needs, with emphasis on agricultural resource management and productivity in Africa and South Asia. Lundy et al. (2015) and Pittelkow et al. (2015a) however did not include treatments in which full tillage was practiced with residue removal. Rather, they applied a control treatment in which residues were incorporated into the soil during tillage. This was done in order to isolate yield responses to tillage alone in comparison to NT with or without residues. Although subtle, such standardization illustrates an important point regarding the choices researchers make in defining crop management practices for treatments and their implications when extrapolating and discussing meta-analytical results. While only including tillage treatments with residue incorporation establishes systems with similar residue input levels, it arguably poorly reflects farmers’ predominant practices in mixed crop-livestock farming systems – especially in sub-Saharan Africa and South Asia – in which residues tend to be exported from fields for feed, fuel, housing materials or other purposes (Erenstein, 2002; Valbuena et al., 2012). As such, the applicability of meta-analytical results to smallholder farming conditions in either sub-Saharan Africa and South Asia may be questioned.

Given the large variation in crop management practices that result from differences in the scale of farming operations, the nature of farm enterprises (e.g., crop-based or mixed crop-livestock farming systems) and cropping patterns in different farming systems, one may therefore ask: Does the presentation of average results from ‘global meta-analyses’ in agronomy make sense? Our case studies show the ways in which the practical value of meta-analyses (and manipulative experiments) to provide comprehensive evidence on topics of development relevance is undermined by the social construction of treatment categories that may be decoupled from the conditions faced by farmers themselves.

### Results extrapolation and the complexity of agricultural systems

Most meta-analyses reviewed in this study used primary data from small-plot agronomic trials. The problems associated with extrapolating results from small plot experiments to whole fields, cropping systems (in which crops are often rotated) and farming systems have however been widely acknowledged (Cassman, 2007; Doberman, 2012; Kravchenko et al., 2017). These problems also affect meta-analysis. Farmers are typically time and often resource constrained. Many manage multiple separate fields each of which may be environmentally heterogeneous – across landscapes. Farmers may therefore not be able to rigorously and evenly implement recommended crop management practices across fields and farm units with the same precision as researchers managing small-plot trials. This therefore casts some doubt about the usefulness of data from small-plot trials. Kravchenko et al. (2017), for example, demonstrated that yield results from small-plot OA experiments were not always consistent with field-scale measurements of the same treatments. Caution is therefore needed when extrapolating results from small-plot research to the field, farming system, landscape and global levels.

These problems are most apparent in the OA case study. Badgley et al. (2007a), for example, extrapolated OA yield responses from plot studies to the global agricultural system, concluding that OA could feed the world’s population with nitrogen requirements supplied in situ by legumes, without expanding the footprint of agriculture. Connor (2008) conversely pointed out that soil moisture deficits would likely constrain the productivity of legumes in arid environments. He also noted that rotations with legumes may also not be feasible where legumes are less profitable or important than other crops for income generation and food production. Assessing productivity on a yield per unit of time basis, rather than yield alone, may therefore be an appropriate alternative in such comparisons (Kirchmann et al., 2016).

Leifeld (2016) also referenced landscape-scale considerations when contesting data presented by Ponisio et al. (2015). He contended that OA is unable to cope with high-fecundity and rapidly dispersing pests, which could result yield losses more severe than observed in isolated, small-plot experiments. Leifeld (2016) also evoked ‘Borlaug hypothesis’ arguments that low-yielding farming systems may require the conversion of natural ecosystems to meet expanding food demand, thereby negatively affecting biodiversity. Ponisio and Kremen (2016) countered with evidence of the positive effects of organic and ecologically managed farmland on pest suppression at the landscape scale. They also highlighted the study of Meyfroidt et al. (2014), who showed that higher yields and profitability can also drive agricultural expansion and deforestation under conventional practices.

Considering the complexity of these problems, Brandt et al. (2013) proposed that bias could be reduced and science quality increased if researchers using metaanalysis make their research protocols and intended methods (including the scale at which results will be interpreted and extrapolated) publically available, for example, through online posting or journal publication, prior to undertaking meta-analysis. ‘Pre-registration’ of planned studies may be a logical suggestion (Gurevitch et al., 2018), though it implies serious changes in research practice and re-thinking of how journals accept papers and conduct peer-review. This proposition has therefore not yet been widely applied in agronomy or other disciplines. While there is no easy answer to how to rectify this conundrum, our review presents and important step in challenging underlying assumptions that meta-analysis can provide definitive and unifying conclusions as proposed by Garg et al. (2008), Borenstein et al. (2009), Rosenthal and Schisterman (2010) and Fisher (2015).

## CONCLUSIONS

In this paper, we reviewed prominent meta-analyses published since 2007 on two of the most widely debated topics in contemporary agronomic and cropping systems research: OA and CA. Adopting a political agronomy framework that recognizes the ways in which agricultural research is socially and politically situated, we analysed these studies considering three methodological and epistemological concerns. The first focused on the influence of scientific and agricultural paradigm on the ways in which agronomists categorize cropping systems in experimental and meta-analytical comparisons. The second concern considered the justification for and conceptual framing of research questions in OA and CA meta-analyses. Last, we discussed the ways in which the extrapolation of meta-analytical research results from small experimental plots to whole farming systems and even global scales appears to generate rather than resolve debate.

Our case studies indicate that scientists designing meta-analyses in agronomy grapple with challenges related to the simplification of farmers’ complex and variable crop management practices into categories that can be analysed statistically. This simplification –which also involves subjective decision making to include or exclude treatments and management regimes – is not inherent to meta-analysis alone. Rather, these issues influence the design and administration of agronomic experiments in general. Yet, the problems of standardization and simplification appear to be amplified by meta-analyses, at times reducing their value for agricultural policy or improving farmer practice.

The framing of meta-analysis is an important yet politically contested topic. Most meta-analyses reviewed made a point of highlighting the size and comprehensiveness of their datasets, while implying a capability to answer questions of regional or ‘global’ significance for food production, food security, or environmental challenges. The potential of these analyses to achieve unifying conclusions that have global as well as local relevance, however, ironically appears to be undermined by the large geographic scale at which results tend to be presented. The presentation of ‘global’ average results that are decoupled from the context-specific and diverse qualities of farming systems is unlikely to meaningfully inform policy and investment decisions, nor inform ways to improve farmer practice. The debates described in our case studies also point to the crucial importance of analytical scale. Small-plot and research station-based experiments may not be representative of whole field or whole farm functioning, and may inadequately reflect cropping system dynamics and the economic and resource allocation choices made by farmers outside the experimental setting. This problem is inherent to the organization of agronomic research, and appears to be amplified in meta-analysis that combines multiple field trial studies to generate more comprehensive results.

Our case studies indicate that meta-analyses have not been able to reduce controversies within agronomy – in some cases, they to do just the opposite. This paper therefore represents an important first step towards rethinking the position of meta-analysis in agronomy. While meta-analysis is increasingly popular and is of general scientific interest, we suggest that its use to appraise and to prioritize agricultural research and development investments should be carefully tempered by consideration of the method’s analytical limitations. Scientists and policy makers evaluating the results of future meta-analyses should consider how treatments are defined and constructed, and how papers and data are collected, screened and analysed. Although most assessments of the value of meta-analysis focus on quantitative methods, the ways in which researchers justify, frame and position their research questions are also important, as these factors can condition the ways in which statistical analyses are interpreted and discussed. In addition, critical evaluation of the ways in which researchers interpret data derived from plot-scale experiments and discuss their results in the context of diverse farming systems and at regional or global scales is needed. Lastly, when meta-analysis is applied to topics that are highly politicized, as is the case with OA and CA, more cautious interpretation of results that recognizes the socially and politically embedded nature of agricultural research is required.

## Footnotes

1 The philanthropist Howard Buffet (2015) submitted a comment stating that ‘You can listen to the academics or you can listen to the farmers’ (p. 1), while supplying qualitative ‘success stories’ of African CA farmers as counter-evidence for CA’s benefits.

2 Peer-review comments on the work of Knapp et al. (2018) are particularly germane to this issue. Readers are referred to Reviewer #1’s comments in the downloadable Peer Review File available online at https://www.nature.com/articles/s41467-018-05956-1
